# The *agr* Inhibitors Solonamide B and Analogues Alter Immune Responses to *Staphylococccus aureus* but Do Not Exhibit Adverse Effects on Immune Cell Functions

**DOI:** 10.1371/journal.pone.0145618

**Published:** 2016-01-05

**Authors:** Mara Baldry, Betül Kitir, Hanne Frøkiær, Simon B. Christensen, Nico Taverne, Marjolein Meijerink, Henrik Franzyk, Christian A. Olsen, Jerry M. Wells, Hanne Ingmer

**Affiliations:** 1 Department of Veterinary Disease Biology, Faculty of Health and Medical Sciences, University of Copenhagen, Frederiksberg C, Denmark; 2 Department of Drug Design and Pharmacology, University of Copenhagen, Copenhagen, Denmark; 3 Center for Biopharmaceuticals, University of Copenhagen, Copenhagen, Denmark; 4 Host Microbes Interactomics Group, Wageningen University, Wageningen, The Netherlands; Universitätsklinikum Hamburg-Eppendorf, GERMANY

## Abstract

*Staphylococcus aureus* infections are becoming increasingly difficult to treat due to antibiotic resistance with the community-associated methicillin-resistant *S*. *aureus* (CA-MRSA) strains such as USA300 being of particular concern. The inhibition of bacterial virulence has been proposed as an alternative approach to treat multi-drug resistant pathogens. One interesting anti-virulence target is the *agr* quorum-sensing system, which regulates virulence of CA-MRSA in response to *agr*-encoded autoinducing peptides. Agr regulation confines exotoxin production to the stationary growth phase with concomitant repression of surface-expressed adhesins. Solonamide B, a non-ribosomal depsipeptide of marine bacterial origin, was recently identified as a putative anti-virulence compound that markedly reduced expression of α-hemolysin and phenol-soluble modulins. To further strengthen solonamide anti-virulence candidacy, we report the chemical synthesis of solonamide analogues, investigation of structure–function relationships, and assessment of their potential to modulate immune cell functions. We found that structural differences between solonamide analogues confer significant differences in interference with *agr*, while immune cell activity and integrity is generally not affected. Furthermore, treatment of *S*. *aureus* with selected solonamides was found to only marginally influence the interaction with fibronectin and biofilm formation, thus addressing the concern that application of compounds inducing an *agr*-negative state may have adverse interactions with host factors in favor of host colonization.

## Introduction

*Staphylococcus aureus* is a colonizer of the human nasal cavity and skin in around 20–30% of the healthy human population and yet, it is a notorious opportunistic pathogen, causing severe community-associated and nosocomial infections [1,2]. Infections caused by *S*. *aureus* range from mild superficial skin infections, to toxinosis or toxic shock syndrome, and severe systemic life-threatening conditions such as endocarditis or meningitis [2]. *S*. *aureus* can possess a wide repertoire of acquired resistance genes, including methicillin resistance (i.e MRSA) which limits treatment options [3]. A key regulator of virulence gene expression is the accessory gene regulator (*agr*) quorum-sensing system, which is highly active in the CA-MRSA USA300 strain, one of the most prevalent and virulent culprits of community-associated infections [3,4]. Activation of the *agr* two-component system occurs in response to the accumulation of self-produced cyclic thiolactone peptides also known as autoinducing peptides (AIPs). AIPs bind to the AgrC histidine kinase of the *agr* two-component system and stimulate the expression of a regulatory RNA designated RNAIII, the effector molecule of *agr* signaling [4]. At high cell densities AIP accumulation is responsible for up-regulating expression of exoproteins including the *hla-*encoded α-hemolysin, a prominent virulence factor in *S*. *aureus*, and down-regulating expression of surface-associated proteins (such as *spa-*encoded protein A) [5,6]. Conversely, at low cell densities exoprotein production is repressed and surface proteins involved in interactions with host factors are highly expressed [7,8]. There are at least four classes of AIPs in *S*. *aureus* each differing slightly in their chemical signaling through a cognate AgrC receptor and displaying antagonism in strains harboring other classes of AIPs [7]. Synthetic analogues of AIPs can also inhibit MRSA virulence, and naturally occurring antagonists of this system have been identified as well [8,9]. Recently, two novel cyclodepsipeptides, named solonamide A (**1**) and B (**2**), were isolated from a marine bacterium (*Photobacterium* spp. strain S2753) with structures remarkably similar to those of the AIPs [9,10]. They competitively inhibit *agr* by interfering with the binding of AIPs to the *agr* sensor kinase, AgrC [10]. Interference with bacterial virulence and/or cell-to-cell signaling pathways by solonamides may be a useful strategy for therapy against *S*. *aureus* infections. Such anti-virulence approaches will inherently exert less selective pressure towards development of bacterial resistance as compared to antibiotics, and importantly they rely on a robust host immune response for the ultimate clearance of the infection [11]. In this study, we investigated the importance of solonamide B and solonamide analogue structure in fine tuning the *agr* response. Furthermore, we addressed the concern that these anti-virulence compounds might influence factors that promote host colonization, or have adverse effects on host immune responses.

## Materials and Methods

### Bacterial strains and growth conditions

Strains used in this study, and their sources are listed in Table 1. For preparations of live or UV inactivated S. aureus samples specifically, overnight cultures were diluted 1/100 in fresh warm TSB, incubated at 37°C while shaking at 200 rpm, and upon reaching OD_600_ 0.5 test compounds in vehicle or pure vehicle were added to give a final concentration of 10 μg/mL. Cultures were grown to an OD_600_ 1.7 and spun down. The supernatants were collected and frozen in 1 mL aliquots and the bacterial pellets were washed twice in sterile phosphate buffered saline solution (PBS). Washed bacteria were adjusted to OD_600_ 0.5 in 20 mL PBS and 10 mL were frozen directly in 1 mL aliquots and the remaining 10 mL were subjected to UV radiation (*λ* = 254 nm; CL-1000 cross-linker; UVP, Cambridge, United Kingdom) by pulsed UV radiation of 6 sec per pulse for a total of 90 sec. Samples prior to UV and after UV were plated on TSA for number of colony-forming unit (CFU) analysis and multiplicity of infection (MOI) calculation, as well as for checking the viability after UV-irradiation. The Gram-positive bacteria Lactobacillus acidophilus NCFM (Danisco, Copenhagen, Denmark), and the Gram-negative bacteria Escherichia coli Nissle 1917 O6:K5:H1 (Statens Serum Institut, Copenhagen, Denmark) were grown as previously described [12].

**Table 1:** Strains and their sources.

Strain	Description	Source / Reference
PC322	*S*. *aureus* 8325–4 *hla*:*lacZ*	S.J.Foster, [13]
PC203	*S*. *aureus* 8325–4 *spa*:*lacZ*	S.J.Foster, [13]
SH101F7	*S*. *aureus* 8325–4 *rnaIII*:*lacZ*	S.J.Foster, [13]
NCTC 8325–4	*S*. *aureus* WT (*agr group I*)	S.J.Foster, [13]
RN6607	*S*. *aureus* (*agr group II*)	R. Novick [14]
RN4850	*S*. *aureus* (*agr group IV*)	R. Novick, [14]
M0Z53	*S*. *aureus* (*agr group III*)	R. Novick, [14]
RN6911	*S*. *aureus* (8325–4 Δ*agr)*	R. Novick, [15]
FPR3757	CA-MRSA USA 300	ATCC Boras,Sweden
RN10829	*S*. *aureus* AgrC-I-WT P3::*blaZ*	R. Novick, [16]
NCFM	*Lactobacillus acidophilus*	Danisco, CPH, DK
*E*. *coli* Nissle 1917	O6:K5:H1	SSI, CPH, DK
DU1090	*S*. *aureus* (8325–4 Δ*hla)*	O’Reilly [17]
WCFS1	*L*. *plantarum* WCFS1	NIZO Food Research, Ede, NL [18]

### Synthesis of modified solonamide analogues

For the synthesis of analogues SolB-NaI (**5**) polystyrene 2-chlorotrityl chloride resin was added to a fritted syringe and swelled in dry CH_2_Cl_2_. A solution of Fmoc-L-Leu-OH (128 mg, 0.35 mmol, 2.5 equiv) and *i-*PrNEt_2_ (0.12 ml, 0.7 mmol, 5 equiv) in dry CH_2_Cl_2_ was added and the resin loading was allowed to proceed on a rocking table for 1h. After washing with CH_2_Cl_2_ (×3) the resin was capped with CH_2_Cl_2_–MeOH–*i-*PrNEt_2_ (7:2:1) for 30 min. The resin was then washed with DMF (×3), MeOH (×3), and CH_2_Cl_2_ (×3). The Fmoc group was removed with piperidine–DMF (1:4, 4 ml, 2 × 30 min) and DBU–piperidine–DMF (2:2:96, 4 ml, 30 min), and the resin was then washed as described above. Fmoc-D-Ala-OH (139 mg, 0.42 mmol, 3 equiv) in DMF (3 mL) was pre-incubated with 2, 6-lutidine (98 μl, 0.84 mmol, 6 equiv) and HATU (157 mg, 0.41 mmol, 2.95 equiv) for 10 min before addition to the resin and the reaction was allowed to proceed on a rocking table for 24 h. After this washing procedure the Fmoc group was removed with piperidine–DMF (1:4, 4 ml, 2 × 30 min) and DBU–piperidine–DMF (2:2:96, 4 ml, 30 min), and the resin was washed with DMF (×3), MeOH (×3) and CH_2_Cl_2_ (×3). The next two amino acids in the sequence (Fmoc-D-Leu-OH and Fmoc-L-Nal-OH) were introduced according to the same procedure. A portion of the polystyrene 2-chlorotrityl-bound Fmoc-L-NaI-D-Leu-D-Ala-L-Leu (50 mg, 0.027 mmol) was transferred to fresh fritted syringe and swelled with CH_2_Cl_2_ before the standard Fmoc group removal and washing as described above. Then β-hydroxyoctanoic acid (7 mg, 0.054 mmol, 2 equiv) was pre-incubated for 10 min with DIC (8 μl, 0.053 mmol, 1.95 equiv) and HOBt (7 mg, 0.053 mmol, 1.95 equiv) and added to the resin. The reaction was allowed to proceed on a rocking table for 24 h and the resin was washed with DMF (×3), MeOH (×3) and CH_2_Cl_2_ (×3), and then it was treated with TFA–CH_2_Cl_2_ (1:1, 2 mL, 2 × 30 min) followed by washing with CH_2_Cl_2_ (3 ×2 mL) and all fractions were pooled in a round-bottomed flask and concentrated under reduced pressure. Co-evaporation with toluene (×2), toluene–CH_2_Cl_2_ (1:1, ×2), and hexane–CH_2_Cl_2_ (1:1, ×2) afforded the crude pentamer (18 mg), which was used directly for macrolactonization in solution under high dilution. Thus, *i-*PrNEt_2_ (21 μl, 0.11 mmol, 4 equiv), DMAP (5.5 mg, 0.042 mmol, 1.5 equiv), and HATU (22.6 mg, 0.042 mmol, 1.5 equiv) dissolved in DMF (2 mL) were added to a stirred solution of linear crude (18 mg, 0.028 mmol) in DMF (60 mL). After stirring at ambient temperature for 16 h, the reaction mixture was diluted with EtOAc (50 mL), washed with 1 M HCl (50 mL), dried (NaSO_4_), filtered, and concentrated *in vacuo*. The residue was purified with complete separation of the two diastereosiomers by preparative HPLC. Compound (**5)** was obtained as white fluffy solid (2.4 mg, 13%). ^1^H NMR (400 MHz, DMSO-*d*_6_) δ 8.23 (d, *J* = 8.3, 1H), 8.21 (d, *J* = 7.2, 1H), 8.13 (d, *J* = 8.0, 1H), 7.99 (d, *J* = 7, 1H), 7.98 (d, *J* = 7, 1H), 7.89 (d, *J* = 8, 1H), 7.77 (m, 1H), 7.57 (dt, *J* = 6.6, 13.6, 1H), 7.51 (dt, *J* = 6.6, 13.6, 1H), 7.39 (m, 2H), 4.70 (m, 1H), 4.66 (m, 1H), 4.24 (t, *J* = 7.4, 1H), 4.20 (m, 1H), 3.68 (m, 1H), 3.43 (m, 1H), 3.23 (m, 1H), 2.20 (dd, *J =* 16.5, 7.8, 1H), 2.13 (dd, *J* = 14.1 6.4), 1.53–1.17 (m, 17H), 0.86 (m, 6H), 0.83 (m, 3H), 0.72, (d, *J* = 6.9, 3H), 0.68 (d, *J* = 6.9, 3H); ^13^C NMR (100 MHz, DMSO-*d*_6_) δ 171.6 (2C), 171.1 (2C), 171.0, 133.4, 131.8 (2C), 128.4, 127.4, 127.0, 125.8, 125.3, 125.2, 123.9, 88.4, 67.5, 53.9, 50.9, 48.3, 46.3, 43.3, 40.8, 40.5, 36.6, 31.3(2C), 23.9, 23.6, 23.4, 23.3, 22.3, 21.7, 21.5, 18.3, 14.1; HRMS: calcd for [M + H]^+^ 637.3887, found 637.3892; ΔM = 0.8 ppm.

For the synthesis of the lactam analogues Am15-D (**6**), Am15-L (**7**), Am16-D (**8**), and Am16-L (**9**); 2-Chlorotrityl resin (1.6 mmol/g; 0.250 g, 0.4 mmol) was transferred to a Teflon reactor (10 mL) in which it was swelled in dry DCM (5 mL) and then treated with 10% DIPEA in dry DCM (5 mL) for 2 min, and then washed twice with dry DCM (each 5 mL for 2 min). The appropriate Fmoc-aa-OH building block (2 equiv, 0.8 mmol) in dry DCM (4.5 mL) containing DIPEA (4 equiv, 0.28 mL) was added to the resin and then shaken for 1 h. Then the resin was capped with DCM–MeOH–DIPEA (80:15:5, 3 mL, 2 × 5 min). The Fmoc group was removed with 20% piperidine in DMF (5 mL, 2 × 10 min), and then the resin was washed sequentially with DMF, MeOH and DCM (each 3 × 3 min with 5 mL). The following amino acid building block (4 equiv) was coupled with PyBOP (4 equiv) and DIPEA (8 equiv) in dry DMF (4.5 mL) for >2 hours. The pentameric linear intermediates were assembled by repetition of this Fmoc de-protection and coupling cycle, and were then cleaved from the resin with 50% TFA-DCM (3 × 30 min each with 3 mL). The filtrate was co-evaporated with toluene (3 ×), and the resulting residue purified by preparative HPLC (column: Phenomenex Luna C18(2), 5 μm, 21.2 × 250 mm) using a gradient of 20% → 50% eluent B during 20 min (A: H_2_O–MeCN–TFA 95:5:0.1; B: H_2_O–MeCN–TFA 95:95:0.1) to give the linear peptides in yields of 25–50%. Cyclization of the linear intermediates (0.02–0.03 mmol) was perfomed by dissolution in DMF (1–2 mL), and then this solution was added dropwise to a solution of TBTU (6 equiv), HOAt (6 equiv) and DIPEA (12 equiv) in DMF–DCM (1:6; 10 mL). The mixture was stirred for 16 h, after which the DCM was removed *in vacuo*, and then the residue was purified by preparative HPLC (as above) with a gradient of 30% → 95% B during 20 min to give the pure analogues in 35–65% yield. The target compounds were characterized by analytical HPLC (column: Phenomenex Luna C18(2), 3 μm, 4.6 × 250 mm) using the same eluents A and B as for preparative HPLC, as well asby HRMS for which spectra were obtained by using a Bruker MicroTOF-Q II MS detector. The analyses were performed as ESI-MS (m/z): [M+H]^+^.

*Am15-D* (**6**). Analytical HPLC (20% → 100% B during 30 min): *t*_R_ = 26.60 min. HRMS: calcd for [M + H]^+^ 586.3968, found 586.3939; ΔM = 4.9 ppm.

*Am15-L* (**7**). Analytical HPLC (20% → 100% B during 30 min): *t*_R_ = 28.65 min. HRMS: calcd for [M + H]^+^ 586.3968, found 586.3969; ΔM = 0.1 ppm.

Synthesis of solonamides A and B (SolA (**1**) and SolB (**2**)), as well as epi-solonamides A and B (ESA (**3**) and ESB (**4**)) was carried out as described by Kitir et al [19].

### Agar Diffusion Reporter Assay

The reporter assay was conducted as described by Nielsen et al. [20] Test compounds in DMSO, supernatants of strains 8325–4 (AIP-I) and M0Z53 (AIP-III), as well as H_2_O were used as controls. Incubation until blue color appeared in plates varied from 9 h to 48 h.

### Activity of solonamides in WT and AgrC reporter strains using the β-lactamase assay

The method used is described by Nielsen et al. 2014 [10]. Briefly, 10 μg/mL of the solonamides and their analogues (final concentration), or DMSO (solvent), and 1/10 volume of spent medium containing or free from AIP-I were used. The β-lactamase activity of the samples was subsequently determined by using the nitrocefin hydrolysis method as described by Ji et al. [14]. Statistical analysis was performed using the Student’s t-test (2-tailed).

### Fibronectin-binding assay

Untreated 96-well plates (Nunc 265301) were incubated with 100 μL per well of 10 μg/mL fibronectin from human plasma for 24 h while shaking at 4°C. The plates were then washed three times with 1% bovine serum albumin in phosphate-buffered saline (PBS). *S*. *aureus* strains 8325–4 (WT) and USA300 were grown with 10 μg/mL of ESB **(4),** Am16-L (**9**), or vehicle (DMSO) from OD_600_ 0.5 after inoculation into fresh TSB from an overnight culture. RN6911 (*Δagr*) was also included. Samples were harvested at OD_600_ 1.7 and added to the Fibronectin-coated wells and incubated for 1 h statically at 37°C. After washing, the attached cells were fixed with 2.5% glutaraldehyde in PBS for 1 h statically at 37°C and stained with 0.1% crystal violet for 30 min at room temperature, washed three times with water, and quantified by resuspension in acidified ethanol and measured at 570 nm. Significance between samples was calculated using the Student’s t-test (2-tailed).

### Static biofilm assay

A starter culture of each strain was grown in TSB to an OD_600_ 0.5. From this culture 50 μL was withdrawn and diluted 10-fold (from 10^−1^ to 10^−5^) in 0.9% NaCl solution. 5 μL of each dilution was inoculated into 200 μL TSB. Compound in DMSO was added to each respective well to a final concentration range of 5, 10, 20, 40 and 80 μg/mL. DMSO and no cells were used as controls. The microtiter plates were incubated for approximately 20 h at 37°C without shaking. The biofilm was then washed twice with 0.9% NaCl (200 μL), dried in a LAF bench, stained with 125 μL crystal violet (0.1%) for 30 min, followed by a 3× final wash with 200 μL 0.9% NaCl. To quantify the biofilm formation the stained biofilm was solubilized in 200 μL 95% ethanol, of which 100 μL was transferred to a new microtiter plate and the absorbance measured at 590 nm.

### Murine dendritic cell (DC) isolation and stimulation

All animals used as a source of bone marrow cells were housed under conditions approved by the Danish Animal Experiments Inspectorate (Forsøgdyrstilsynet) according to The Danish Animal Experimentation Act; LBK no. 474 from 15/05/2014, and experiments were carried out in accordance with the guidelines of ‘The Council of Europe Convention European Treaty Series (ETS) 123 on the Protection of Vertebrate Animals used for Experimental and other Scientific Purposes’. The source of bone marrow cells was female 4–6 month old C57/Black6 –Jtac mice (Taconic, Ejby, Denmark). All mice were sacrificed by cervical dislocation prior to bone marrow extraction for dendritic cell isolation. Dendritic cells were isolated and prepared as previously described by Christensen et al. [21] with no modifications. Naïve DCs (2 × 10^6^ cells/mL) were resuspended in fresh medium and 500 μL/well were seeded in 48-well tissue culture plates (Nunc, Roskilde, Denmark). The stimuli were prepared to give a final volume of 100 μL/well at the following concentrations: 10 μg/mL *L*. *acidophilus* NCFM, and/or 5, 10, and 20 μg/mL of solonamide B, (**2**) epi-solonamide B (**4**) and solonamide analogue Am16-L (**9**), and vehicle (0.1% DMSO). DCs were stimulated alone with *L*.*acidophilus*, solonamides or vehicle, or co-stimulated with bacteria and solonamides. For stimulation with pre-treated and UV-inactivated *S*. *aureus* strains 8325–4, and RN6911 cultures were prepared to a final volume of 100 μL/well at an MOI of 5 (5×10^6^ CFU/mL per well) and added to the DCs.

### Human peripheral blood mononuclear cell (PBMC) isolation and stimulation

The human PBMC assays were approved by Wageningen University Ethical Committee and performed according to the principles of the Declaration of Helsinki. PBMCs were isolated and prepared as previously described [22] with modifications. Peripheral blood of 3 healthy donors, whose written informed consent had been provided, was obtained from the Sanquin Blood Bank, Nijmegen, The Netherlands. Isolated PBMCs were washed and resuspended in Iscove’s Modified Dulbecco’s Medium (IMDM) + glutamax supplemented with 10% heat inactivated Fetal Bovine Serum (FBS), 100 U/mL penicillin and 100 μg/mL streptomycin (Invitrogen, Breda, The Netherlands) to a final concentration of 1×10^6^ cells/mL and 500 μl/well were seeded in 48-well tissue culture plates. For the PBMC stimulation experiment, either compound or vehicle alone were added at a final concentration of 10 μg/mL, or thawed aliquots of the *S*. *aureus* samples adjusted to an MOI of 10 in IMDM without added antibiotics and allowed to adjust for 2 h before adding 50 μL of each sample to the seeded PBMCs. TSB, PBS, IMDM and *L*. *plantarum* WCFS1 were included as controls. The PBMCs were stimulated at 37°C and 5% CO_2_ for 24 h or 4 days. After incubation, the PBMC culture supernatants were collected and frozen in at –20°C until cytokine analysis, and then the cells were harvested and tested for cell viability with Annexin V/PI staining.

### T-cell proliferation assay

Isolated PBMCs were counted and adjusted to a concentration of 1×10^6^ cells/mL. They were then spun down at 300×g for 5 min and the pellet was resuspended in 1 mL sterile PBS + 0.1% BSA containing 50 μg/mL CFDA/SE (Carboxyfluorescein diacetate succinimidyl ester, Cayman Chemicals) and allowed to incubate for 10 min at 37°C. 5 mL IMDM + 10% FBS was added to the cells which were then placed on ice for a further 5 min prior to spinning down and washing 3x with IMDM + 10% FBS. Washed PBMCs were resuspended in complete culture medium to a final concentration of 1×10^6^ cells/mL and 500 μl/well were seeded in 48-well tissue culture plates. PBMCs were then stimulated with the lymphocyte proliferation inducers aCD3 and aCD28 (BD Pharmingen) at concentrations inducing 100% or 25% T-cell proliferation (10 ng/mL and 0.4 ng/mL respectively for aCD3 and 0.6 ng/mL and 0.024 ng/mL respectively for aCD28). PBMCs were then further co-stimulated with either compound or vehicle alone to a final concentration of 10 μg/mL, or thawed aliquots of the *S*. *aureus* samples adjusted to an MOI of 10 in IMDM without added antibiotics and allowed to adjust for 2 h before adding 50 μL of each sample to the seeded PBMCs. TSB, PBS, and IMDM were included as controls. The PBMCs were incubated at 37°C and 5% CO_2_ for 4 days to allow for T-cell proliferation. T-cell proliferation was measured by staining of harvested floating cells with PE-labeled aCD4 according to manufacturer’s instructions and evaluated by flow cytometry using FACS Diva software. Lymphocytes were gated based on the expression of CD4, and the number of cell divisions was gated according to FITC excitation. Significance was tested using one-way ANOVA.

### Cell viability assay

DC, PBMC and T-cell viability was assessed by using the commercially available Annexin V: PI Apoptosis Detection Kit APC (eBiosciences) according to the manufacturer’s instructions. Viability was assessed by flow cytometry and analyzed using FACS Diva software. Significance was tested using one-way ANOVA.

### Cytokine level detection assays

For DCs levels of IL-12, TNF-α, IL-6 and IL-10 (all purchased from R&D Systems, Minneapolis, MN, USA) were detected in culture supernatants by commercially available enzyme-linked immunosorbent assay (ELISA) kits according to the manufacturer’s instructions. For PBMC and T-cell cytokine analysis, cytokines (IL-12, TNF-α, IL-6, IL-10, IL-8 and IL-1β) were measured by BD Cytometric Bead Array Flexset (BD Biosciences) using a FACS CantoII flow cytometer, according to the manufacturer’s instructions and analyzed using the BD FCAP software. Significance was tested using one-way ANOVA.

### Ethical statement

All cells used for the generation DCs were generated from bone marrow cells isolated from mice sacrificed by cervical dislocation. The use of mice was approved by Danish Animal Experiments Inspectorate (Forsøgdyrstilsynet). This is the ethical committee, who approves all animal experiments to be performed in Denmark as well as all the experimental animal facilities in Denmark. All animals used as a source of bone marrow cells were housed under conditions approved by the Danish Animal Experiments Inspectorate (Forsøgdyrstilsynet) according to The Danish Animal Experimentation Act; LBK no. 474 from 15/05/2014, and experiments were carried out in accordance with the guidelines of ‘The Council of Europe Convention European Treaty Series (ETS)123 on the Protection of Vertebrate Animals used for Experimental and other Scientific Purposes. The human PBMC assays were approved by Wageningen University Ethical Committee and performed according to the principles of the Declaration of Helsinki. PBMCs were isolated and prepared as previously described [22] with modifications. Peripheral blood of 3 healthy donors, whose written informed consent had been provided, was obtained from the Sanquin Blood Bank, Nijmegen, The Netherlands

## Results and Discussion

### Synthetic solonamides retain anti-virulence activity while stereochemistry of analogues results in differential *agr* regulation

**Fig 1:**
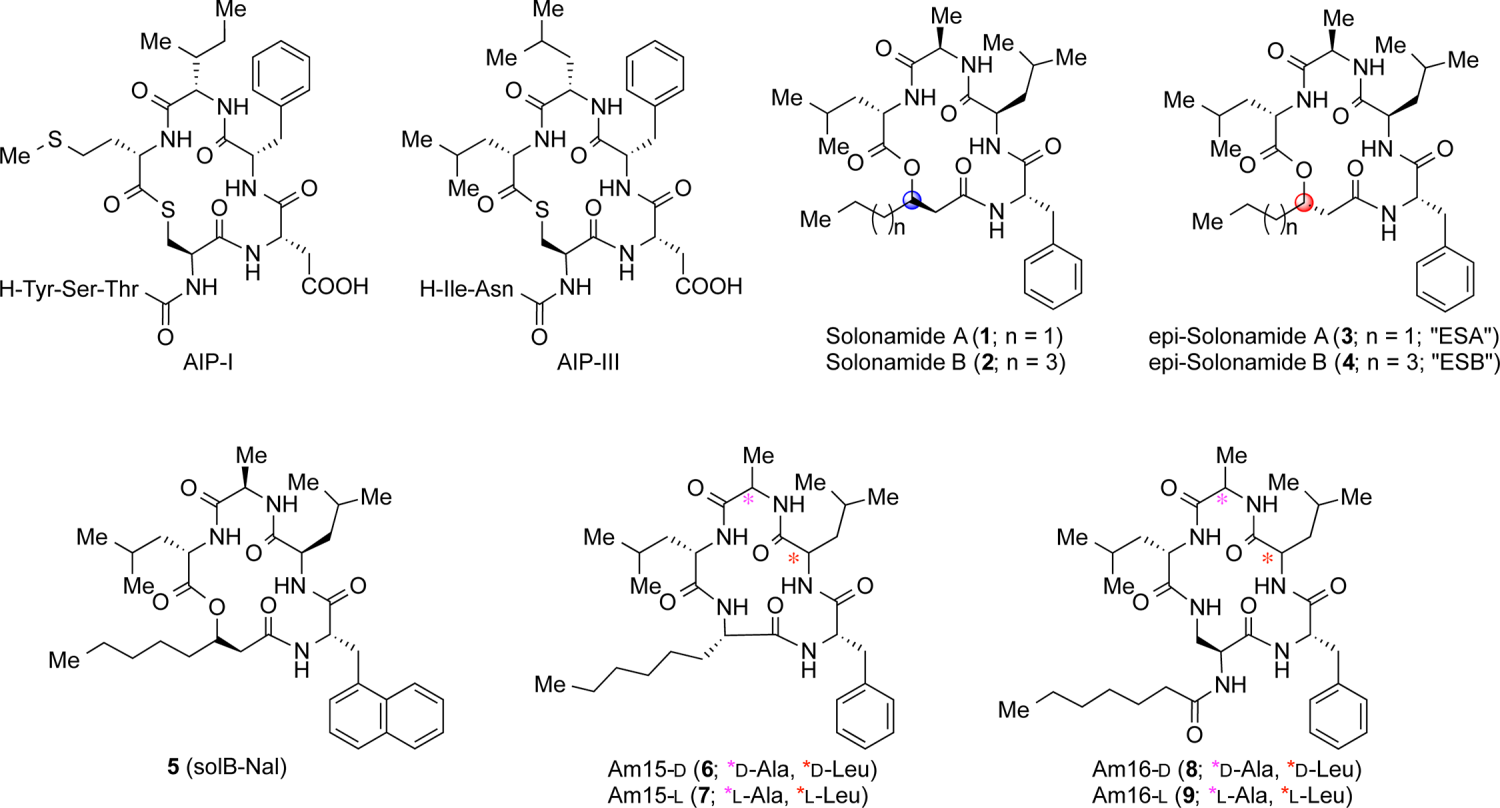
Compound and AIP Structures. Structures of autoinducing peptides (AIP-I and III) used in the study as well as tested depsipeptides and their modified lactam analogues.

To explore the structure–activity relationships of solonamides, total syntheses of solonamides A (**1**) and B (**2**) as well as their β-hydroxy acid epimers [ESA (**3**) and ESB (**4**)] were recently reported [19], and the naphthylalanine analogue SolB-Nal (**5**) was prepared using the same chemistry. The lactam analogues displaying variation in ring size as well as in chirality of selected amino acid residues were assembled by solid-phase peptide synthesis followed by cyclization in solution (vide infra). Structurally, solonamide B closely resembles the natural AIPs as they both contain hydrophobic residues in a cyclic moiety of identical ring size (Fig 1) however; the solonamides contain two D-amino acid residues. To investigate whether a difference in chirality was crucial to obtain efficient *agr* inhibition, we synthesized solonamide-mimicking lactam analogues (**6** and **8**) as well as all-L lactam analogues (**7** and **9**). In analogues **6** and **7**, the ring size was reduced by a single atom to give a 15-membered macrocycle, while the side chain in both types of analogues was similar in length to that of solonamide B.

**Fig 2:**
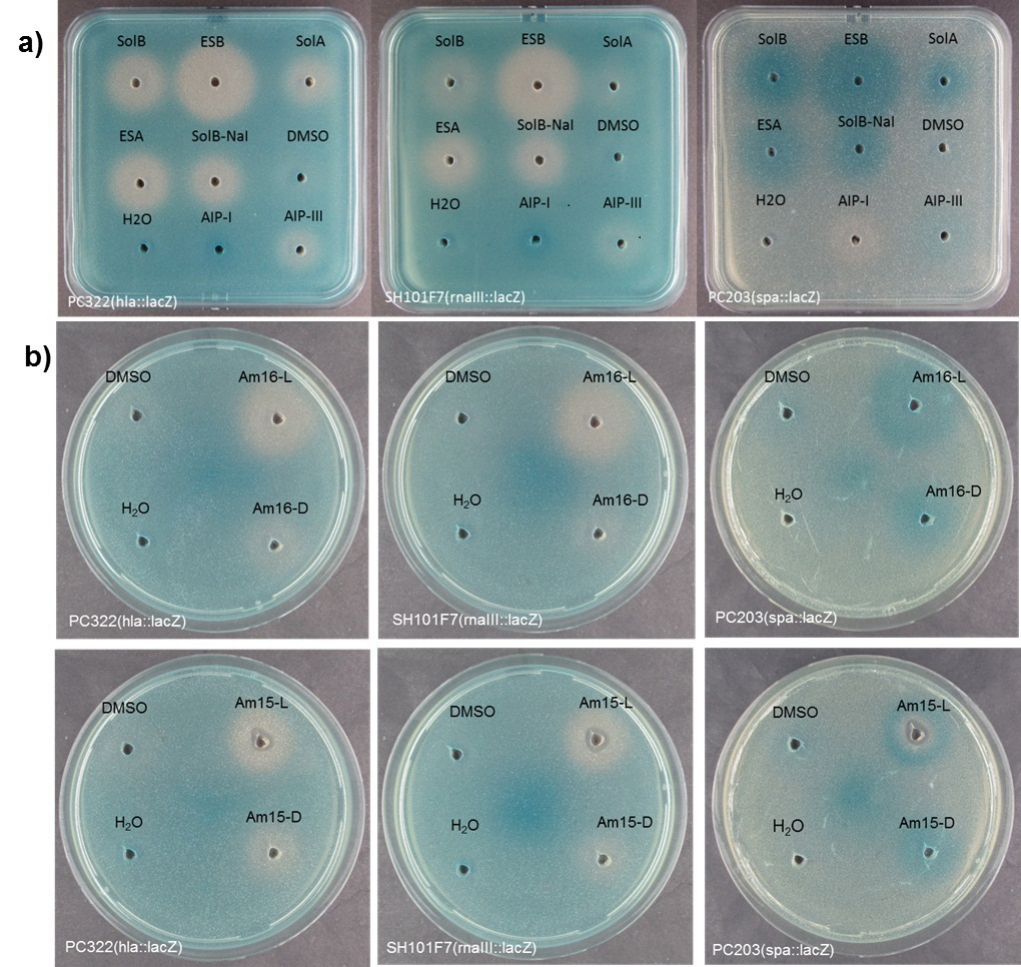
Biological validation of the synthetic solonamides (a) and lactam analogues (b). Agar plates containing the *hla–lacZ* (PC322), the *rnaIII-lacZ* (SH101F7) or *spa–lacZ* (PC203) reporter strains of *S*. *aureus* were exposed to DMSO (20 μL) containing the test compound (0.5 mg/mL). Vehicle (DMSO), H_2_O, AIP-I (autologous) and AIP-III (heterologous) were used as controls. Virulence gene down-regulation is represented by a white zone and up-regulation by a darker than background blue zone.

The ability of synthetic solonamides and lactam analogues to repress *agr* activity was examined by an agar diffusion assay [20] where reporter strains containing the *lacZ* reporter fused to *S*. *aureus* virulence genes *hla* (encoding α-hemolysin), *spa* (encoding Protein A) and the *agr* response regulator gene *rnaIII* were used to measure virulence gene regulation. Here, we confirmed that synthetic solonamide B retained ability to modulate *agr* activity as monitored by increased expression of *spa* and decreased expression of *hla* and *rnaIII* (Fig 2). Furthermore, activity of solonamides is clearly dependent on stereochemical features as the analogues of solonamide B with opposite configuration of the stereocenter in the β-hydroxy acid residue, ESA (**3**) and ESB (**4**), showed markedly larger interference zones than the naturally occurring solonamides, with ESB being the most effective. The lactam analogues also exhibited potent activity, with the most active compounds being those displaying the all-L stereochemical configuration [i.e., Am15-L (**7**) and Am16-L (**9**)] also present in the AIPs.

**Fig 3:**
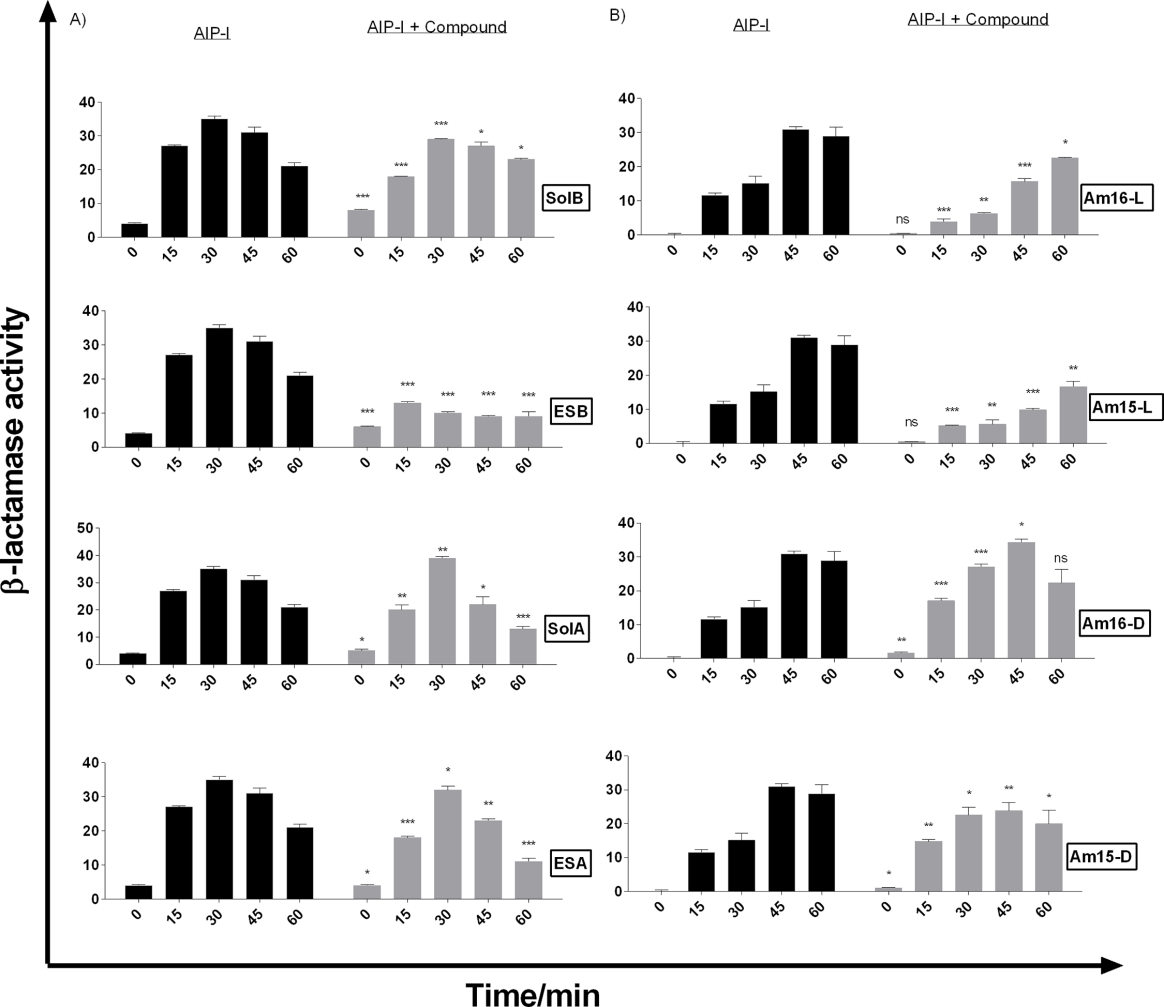
Solonamides (A) and lactam analogues (B) differentially interfere with AgrC activation as monitored by direct RNAIII expression. Cultures of RN10829 (P2-agrA:P3-blaZ) containing the pagrC-I-WT, were grown to an OD_600_ of 0.4–0.5 where a 1/10 volume of AIP-I containing supernatant was added and solonamides and analogues in DMSO to a final concentration of 10 μg/mL. Samples obtained at 15 min time intervals after addition of test solutions were analysed for β-lactamase activity. Each bar represents the average of 3 replicates and the error bars represent the standard deviation. Comparisons were made for each individual time point between AIP and AIP + Compound samples. ns (no significance); *, p<0.05; **, p<0.01; ***, p<0.001.

Interference with the *agr* sensor system was further examined and quantified using the RNAIII-reporter strain RN10829 [10,16]. In accordance with the agar diffusion assay, we observed that all the solonamides showed varying degrees of *agr* inhibition with the most pronounced effect displayed by ESB(**4**), resulting in a three-fold reduction in RNAIII expression in comparison to the control culture induced by AIP (Fig 3A). We found that the lactam analogues Am15-L (**7**) and Am16-L (**9**), which have all-L amino acid configuration, were capable of *agr* inhibition with Am16-L (**9**) almost matching that of ESB (**4**), but with a shorter window of activity (Fig 3A and 3B). Interestingly, the analogues [Am16-D (**8**) and Am15-D (**6**)] containing two D-amino acids as also found in the solonamides, initially displayed marginal activation of *agr* above the level of AIP induction, which overtime reverted back to marginal inhibition. These data highlight the importance of ring size and stereochemistry in fine-tuning the interactions of AIPs, solonamides, and their analogues with AgrC. Based on these investigations solonamide B (**2**), ESB (**4**), and Am16-L (**9**) were selected for further studies.

### Solonamide treatment of *S*. *aureus* does not substantially promote adhesion to host factors or biofilm formation

**Fig 4:**
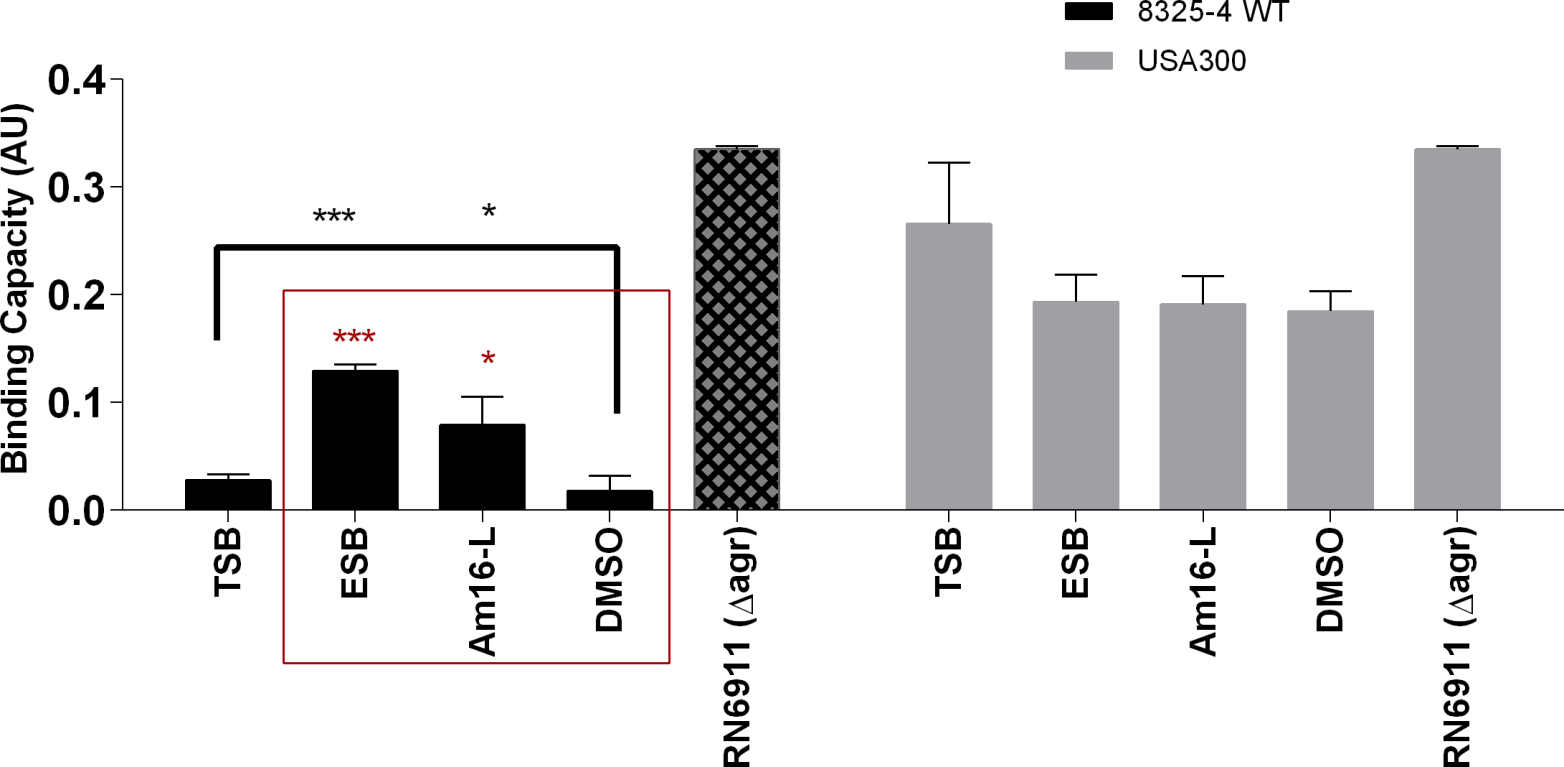
Solonamides marginally enhance adhesion to the extracellular matrix component fibronectin. Upon exposure of *S*. *aureus* strains to 10 μg/mL anti-virulence compounds or vehicle control, attachment to fibronectin was measured in 96-well plates coated with 10 μg/mL fibronectin and staining with 0.1% crystal violet. Absorbance values were corrected for cell density and represented as arbitrary binding units. For statistical significance, comparisons were made between untreated versus vehicle and treated (black bracket), and between vehicle control versus compound treated (red square). ns (no significance);*, p<0.05; **, p<0.01; ***, p<0.001.

**Fig 5:**
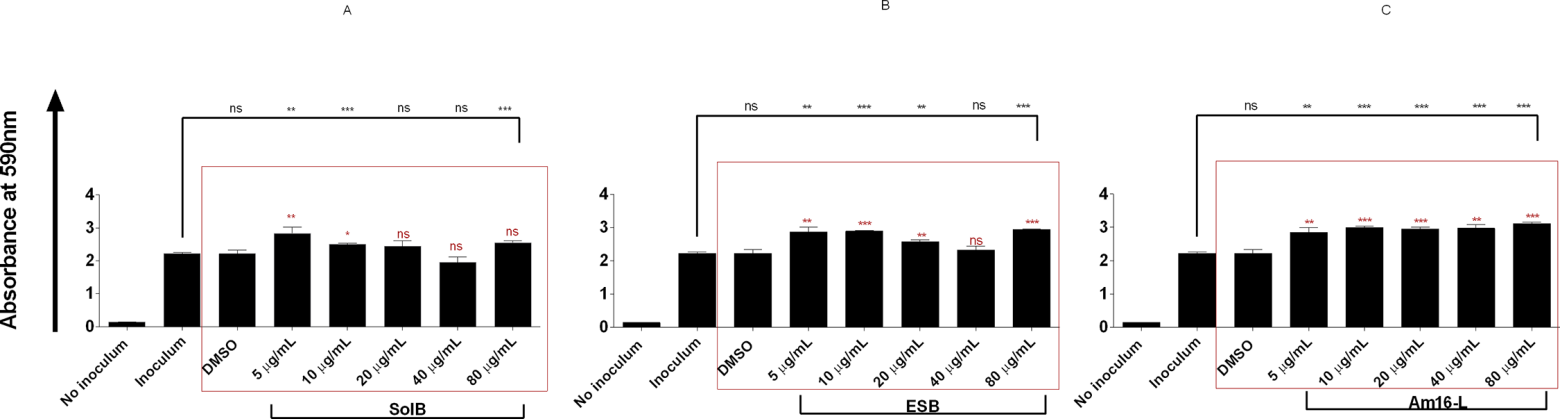
Solonamides marginally enhance biofilm formation of WT strain 8325–4. A dilution series of an 8325–4 culture were inoculated (5 μL) into wells of a 96-well microtiter plate containing 200 μL TSB. SolB (A), ESB (C) or Am16-L (C) was added to final concentrations of 5, 10, 20, 40, and 80μg/mL. Inoculum alone, DMSO and no inoculum were used as controls. Biofilm formation was assessed by crystal violet staining and OD590 nm measurement. Each bar represents the average of 3 experiments, and the error bars represent the standard deviation. For statistical significance, comparisons were made between untreated versus vehicle and treated (black bracket), and between vehicle control versus compound treated (red square). ns (no significance);*, p<0.05; **, p<0.01; ***, p<0.001.

As *agr* balances the expression of exotoxins in the stationary growth phase with the repression of surface-associated adhesins, a key question is whether repression of *agr* by solonamides leads to enhanced stationary phase expression of surface-associated proteins involved in biofilm formation, adhesion and immune evasion such as the *spa* encoded Protein A or the fibronecting-binding protein [23–26]. To address this question we treated *S*. *aureus* strains with our selected solonamides and studied the strains’ capacities to adhere to human fibronectin or to form biofilm. When investigating treated *S*. *aureus* cells we observed that ESB (**4**) (P<0.0001) and Am16-L (**9**) (P<0.02) significantly increased the fibronectin-binding capacity of the 8325–4 WT strain, in comparison to the untreated and vehicle (DMSO)-treated controls, but not to the extent observed in the RN6911 *agr* deletion mutant. This indicates that although an increase in fibronectin binding is observed, it does not mirror an *agr*-negative phenotype. For the CA-MRSA USA300 clinical isolate we observed a slight non-significant and compound-independent decrease in fibronectin-binding capacity (Fig 4). These data suggest that solonamide treatment increases fibronectin binding, which is associated with inactivation of *agr* signaling, but in a strain-dependent fashion. With regards to the influence of treatment on biofilm formation, we observed that solonamide B (**2**), ESB (**4**), and Am16-L (**9**) increased static biofilm in a dose-independent manner; however, the overall increase was marginal (less than 2-fold) as compared to non-treated and vehicle controls (Fig 5A, 5B and 5C). From these results on fibronectin-binding and biofilm formation, we conclude that inhibition of *agr* by the selected anti-virulence compounds only marginally increases binding of *S*. *aureus* to host components without modulating static biofilm formation. These findings alleviate some of the concerns that by interfering with *agr* through the application of our anti-virulence candidates, we significantly promote complications often associated with *agr* negative strains such as: host persistence, colonization and immune evasion.

### Solonamide B (2), ESB (4), and lactam analogue Am16-L (9) are not toxic to immune cells

Solonamide B was previously shown to display undetectable toxicity to both human and bovine neutrophils [10]. To further support these data, we examined the viability of human PBMCs and T-cells as well as murine bone marrow-derived DCs, exposed to our selected compounds, by using the Annexin V/PI staining method. Toxicity testing of our compounds on a wider range of immune cells is imperative given the fact that for any anti-virulence compound to be effective, a healthy immune response towards the disarmed pathogen is needed [6]. We found that none of the tested solonamides nor the lactam analogue at the effective concentration of 10 *μ*g/mL displayed toxicity towards human PBMCs and T-cells, as viability remained above 80% irrespective of incubation period (24 h or 4 days), and was equal to the non-exposed and DMSO-exposed PBMC samples (S1 Fig). Increasing the concentration through a range of 5, 10, 20 and 40 *μ*g/mL over a 4 day incubation period only showed a slight concentration-dependent decrease in viability for solonamide B (**2**) and ESB (**4**) that nevertheless did not drop below 73% at 40 *μ*g/mL (S2 Fig). When examining murine dendritic cells (mDCs) for viability, we observed that after a 20 h incubation period viability ranged from 65% to 71% irrespective of compound exposure (S3 Fig). Collectively, these data imply that our potential anti-virulence compounds are not toxic to immune cells at the concentration that effectively antagonizes *agr* signaling.

### Solonamide-treated *S*. *aureus*, but not solonamides alone, influence immune cell responses

As the selected anti-virulence compounds did not affect viability of human PBMCs, proliferated T-cells or murine DCs, we investigated whether they would modulate cytokine and chemokine responses in unstimulated and microbially stimulated antigen-presenting cells as well as proliferating T cells. Cytokines IL-6, TNF-α, IL-12, and IL-10 were measured for DCs, and for the PBMCs and aCD3 and aCD28 stimulated T-cells, IL-1β and the chemokine IL-8 were also measured. The tested compounds had no effect on the cytokine and chemokine secretion in non-stimulated DCs, PBMCs or T-cells activated with aCD3 and aCD28 compared to untreated controls, indicating that the compounds alone do not stimulate an immune response. Furthermore, the selected solonamides and analogues exerted no immune-modulating effect on cytokine secretion by *L*. *acidophilus*-activated murine dendritic cells, and neither hampered nor enhanced DC function (S4 Fig).

**Fig 6:**
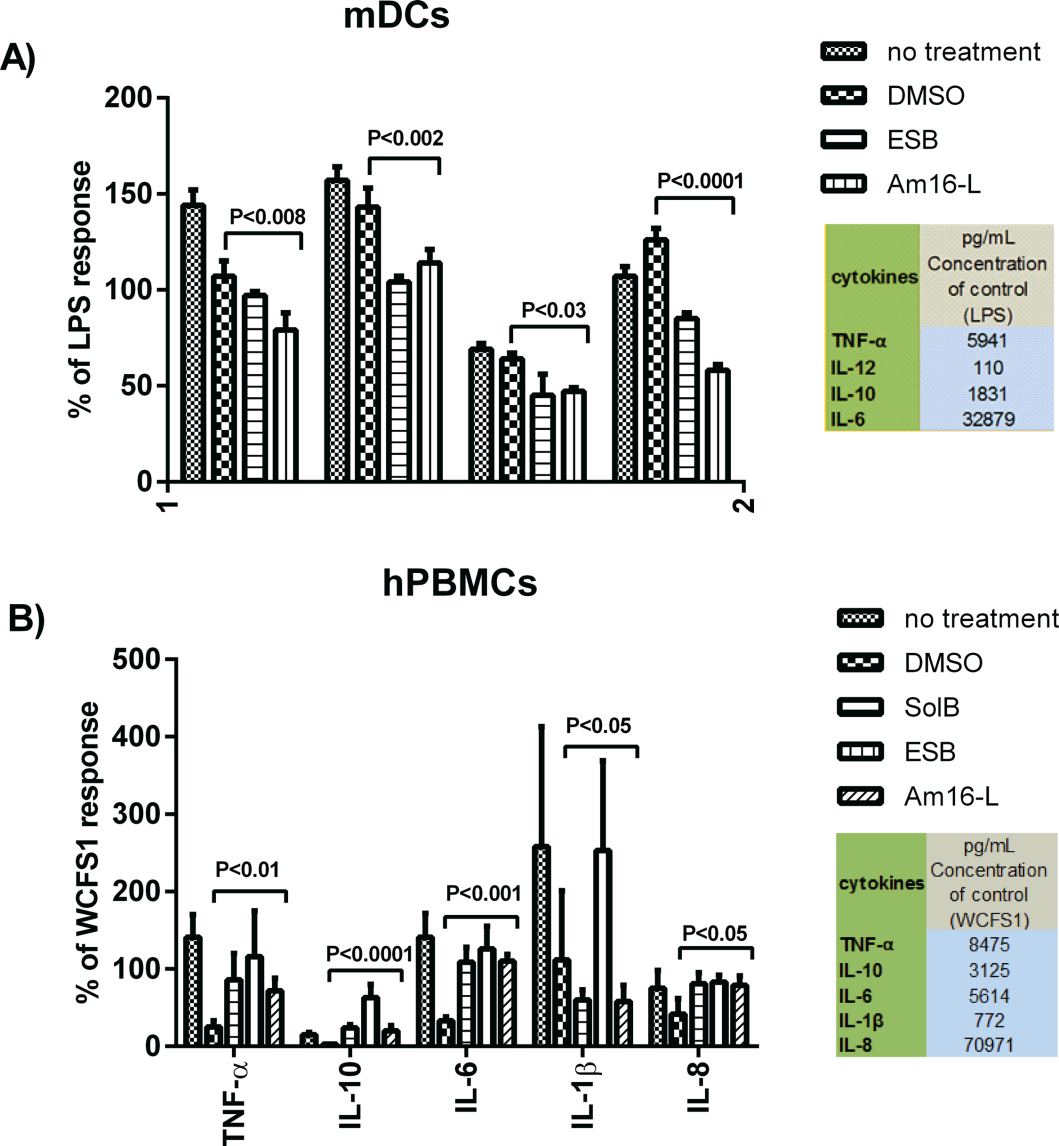
Solonamide-treated *S*. *aureus* influence cytokine production by immune cells. Bone marrow derived murine dendritic cells (A) and human peripheral blood mononuclear cells (B) were stimulated at an MOI of 10 with *S*. *aureus* strain 8325–4 pre-treated with SolB (2) ESB (4), Am16-L (9) or DMSO at 10 μg/mL. Protein concentrations of selected cytokines in the supernatants after 20–24 h incubation were measured by enzyme-linked immunosorbent assay for DCs and by cytometric bead array for human PBMCs. The results are based on 3 biological replicates and are normalized to the stimulus controls (LPS for DC; *L*. *plantarum WCFS1* for PBMCs). Comparisons were made between the DMSO compound vehicle and the compounds.

As *S*. *aureus* harbors a wide repertoire of immune evasion mechanisms [27] we hypothesized that treatment with our *agr*-inhibiting compounds would interfere with some of these mechanisms, and thus allow for a more robust immune response against the treated target pathogen. To investigate this, we exposed DC and PBMCs to compound-treated *S*. *aureus* and measured cytokine responses as indicators of immune modulation and response. We found that the amounts of cytokines secreted by DCs in response to *S*. *aureus* treated with the *agr* inhibitors were significantly lower than those measured with untreated or DMSO treated *S*. *aureus* controls (Fig 6A). In contrast PBMCs incubated with *S*. *aureus* treated with these compounds secreted significantly higher amounts of cytokines compared to DMSO-treated *S*. *aureus* although this was not consistent for IL-1β (Fig 6B). These findings reveal that treatment of *S*. *aureus* with our *agr-*interfering compounds significantly alters the cytokine release pattern depending on the immune cell type.

**Fig 7:**
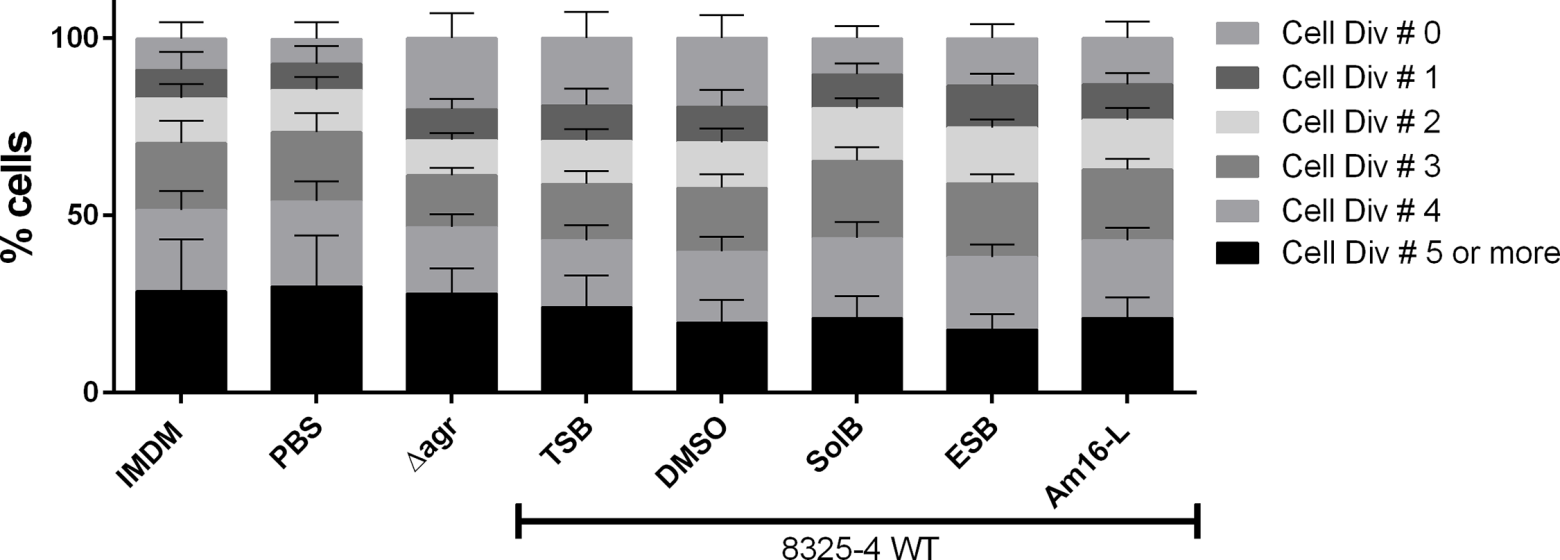
SolB, ESB and Am16-L- reduce interference of T-cell proliferation by *S*. *aureus*. CFDA/SE (25μg/mL) stained human PBMCs (1x10^6^/ml) were co-stimulated with aCD3/aCD28 (0.4ng and 0.024ng/mL respectively) and S. aureus either treated or not with 10μg/mL SolB, ESB or Am16-L at an MOI of 10 for 4 days. Harvested cells were stained with aCD4-PE and analyzed by Flow Cytometry using FACS Diva software. Lymphocytes were gated based on the expression of CD4, and the number of cell divisions was gated according to the CFDA/SE FITC excitation. The data represent the average of 3 donors, and the error bars represent the standard deviation.

One reported mechanism of *S*. *aureus* immune evasion is via the inhibition of T-cell proliferation. A reported mediator of such inhibition is the *S*. *aureus* secretion of the MHC class II analog protein Map, whose expression is regulated by the *agr*-controlled RNAIII regulatory RNA [28,29]. To investigate the possible effects of compound-treated *S*. *aureus* on host immune modulation or evasion, we also examined whether treated bacteria were capable of interfering with T-cell proliferation. We hypothesized that as Map expression is partially controlled by RNAIII, the inhibition of RNAIII via *agr* interference with our compounds could interfere with some of the T-cell proliferation-inhibition properties of *S*. *aureus*. In accordance with the PBMC data, the compounds themselves did not induce T-cell proliferation, nor did they modulate T-cell proliferation in a PBMC population incubated with aCD3/aCD28 to stimulate and expand the T cells. However, when PBMCs were exposed to aCD3/aCD28 in the presence of compound-treated *S*. *aureus* and untreated control strains, we observed that the *Δagr*, non-treated WT strain and the DMSO-treated samples showed a marked increase in non-dividing T cells compared to the PBS, medium control, and compound-treated *S*. *aureus* samples (Fig 7). This observation suggests that the selected solonamides are capable of reducing the partial inhibition of T cell proliferation exerted by WT *S*. *aureus*, thus displaying some degree of modulation. The data also suggests surprisingly that this effect may be *agr*-independent, thus rendering our hypothesis partially void. When the compound-treated *S*. *aureus* strains were UV-inactivated the effect was not observed (S5 Fig) suggesting that inhibition of T cell proliferation by WT *S*. *aureus* might depend on a secreted metabolite or protein, which may not be produced when WT *S*. *aureus* is treated with solonamides.

## Conclusions

In summary our investigations show that solonamide B and analogues harbor no toxicity towards immune cells, and they suggest that no adverse effects are anticipated when using these compounds to target *S*. *aureus* infections. These findings provide important background information for the future *in vivo* characterizations of these compounds as potential safe candidates for anti-virulence therapy targeting *S*. *aureus*.

## Supporting Information

S1 Fig Solonamides and the selected analogue Am16-L do not influence the viability of human PBMCs or proliferated T-cells. Human PBMCS were stimulated with 10 μg/mL solB (2), ESB (4) or the analogue Am16-L (9) for 24 h (A) and 4 days (B) or stimulated for T-Cell proliferation with aCD3/aCD28 and co-stimulated with the selected compounds for 4 days (C) Culture media and DMSO were used as controls. PBMCs and T-cells were harvested; stained with Annexin V/PI, and cell viability measured by flow cytometry. The results are representative of 3 different donors.
(TIF)

S2 Fig Increasing concentrations of solonamides and the selected analogue Am16-L do not influence the viability of human PBMCs. Human PBMCS were stimulated with a concentration gradient of 5, 10, 20 and 40 μg/mL of solB (2), ESB (4) or the analogue Am16-L (9) for 4 days. Culture media and DMSO were used as controls. PBMCs were harvested; stained with Annexin V/PI, and cell viability measured by flow cytometry. The results are representative of 3 different donors.
(TIF)

S3 Fig Solonamides and selected analogues do not influence the viability of naïve or stimulated murine dendritic cells. Bone-marrow-derived DCs were stimulated with *L*. *acidophilus* NCFM (10 **μ**g/mL), solB (**2**), the ESB (**4**) or the analogue Am16-L (**9**) at 20 **μ**g/mL, either alone or in combination with *L*. *acidophilus* NCFM for 20 hrs. DCs were harvested; stained with Annexin V/PI, and then cell viability was measured by flow cytometry. The results are representative of one of 3 reproducible independent experiments.
(TIF)

S4 Fig Solonamides do not exert immunomodulating effects on NCFM-stimulated murine dendritic cells. **(a)** SolB, **(b)** ESB, and **(c)** analogue Am16-L. (Columns 1 = NCFM; 2 = 0.1% DMSO; 3, 4 and 5 = 5, 10 and 20 **μ**g/mL of each compound; 6 = un-stimulated DCs). Bone-marrow-derived dendritic cells (DCs) were co-stimulated *with L*. *acidophilus* NCFM (10 **μ**g/mL) and increasing concentrations of compounds at 5, 10 and 20 **μ**g/mL. Concentrations of IL-6, TNF-α, IL-12 and IL-10 in the supernatants after 20 h were measured by enzyme-linked immunosorbent assay (ELISA). The results are based on at least 3 independent experiments.
(TIF)

S5 Fig SolB, ESB and Am16-L do not reduce interference of T-cell proliferation by UV-inactivated and treated *S*. *aureus*. CFDA/SE (25*μ*g/mL) stained human PBMCs (1x10^6^/ml) were co-stimulated with aCD3/aCD28 (0.4ng and 0.024ng/mL respectively) and either treated or not treated with 10*μ*g/mL SolB, ESB or Am16-L and UV-inactivated *S*. *aureus* at an MOI of 10 for 4 days. 200 *μ*L floating cells were harvested, washed and stained with aCD4-PE for 30 min at 4°C. Cells were further washed and resuspended in FACS buffer. Cells were analyzed by Flow Cytometry using FACS Diva software. Lymphocytes were gated based on the expression of CD4, and number of cell divisions was gated according to the FITC excitation. The data represent the average of 3 donors, and the error bars represent the standard deviation.
(TIF)
